# One-Dimensional Topological Photonic Crystal Mirror Heterostructure for Sensing

**DOI:** 10.3390/nano11081940

**Published:** 2021-07-28

**Authors:** Sayed Elshahat, Israa Abood, Mohamed Saleh M. Esmail, Zhengbiao Ouyang, Cuicui Lu

**Affiliations:** 1 Key Laboratory of Advanced Optoelectronic Quantum Architecture and Measurements of Ministry of Education, Beijing Key Laboratory of Nanophotonics and Ultrafine Optoelectronic Systems, School of Physics, Beijing Institute of Technology, Beijing 100081, China; selshahat@aun.edu.eg; 2 Physics Department, Faculty of Science, Assiut University, Assiut 71516, Egypt; 3 Shenzhen Key Laboratory of Micro-Nano Photonic Information Technology, Key Laboratory of Optoelectronic Devices and Systems of Ministry of Education and Guangdong Province, THz Technical Research Center of Shenzhen University, College of Physics and Optoelectronic Engineering, Shenzhen University, Shenzhen 518060, China; i.abood@szu.edu.cn; 4 Basic Science Department, Faculty of Engineering, Misr University for Science and Technology, Giza 12588, Egypt; mohamed.esmail@must.edu.eg; 5 Collaborative Innovation Center of Light Manipulations and Applications, Shandong Normal University, Jinan 250358, China

**Keywords:** topological photonic crystal, edge-state-mode, electro-optical, sensitivity, quality-factor

## Abstract

A paradigm for high-quality factor (Q) with a substantial fulfillment for appraising sensing ability and performance has been investigated. Through constructing a 1D (one-dimensional) topological photonic crystal (PhC) mirror heterostructure, which is formed by the image view of 1D topological PhC stacking with its original one. In the 1D topological PhC-mirror heterostructure, there is an interesting mode that appeared with the symmetric, typical Lorentzian-line shape with 100% transmittance in the topological mirror edge-state mode (hybrid resonance mode) at the heterostructure interface. Physically, such a mode is a defect mode, but the defect is introduced through topological operations. The high Q-factor of 5.08 × 10^4^ is obtained due to the strong optical localization of the defect mode at the topological edge area. Consequently, this device acts as a narrow passband filter. Moreover, due to the narrow bandpass property, it may be an advantageous reference for many applications in filtering, switching, and sensing. Thus, introducing an electro-optical (EO) polymer layer at the interface to modify the edge defect can tune the defect mode both in frequency and Q-factor for higher spatial pulse compression and higher EO sensitivity. Accordingly, the Q-factor of $10^{5},$ the sensitivity of 616 nm/RIU, and the figure of merit of 49,677.42 RIU^−1^ are obtained. The sensing ability and performance are attributable to the strong optical localization in the interface region and enhanced light-matter interaction. We predict that the 1D topological PhC mirror heterostructure will be an outstanding point in the field of optical sensing, filters, and optical switching in different fields.

## 1. Introduction

In recent times, photonic topological insulators have attracted broad attention from researchers due to their unique properties for edge states that are topologically protected, especially in integrated optics applications [1]. Numerous schemes have been proposed to construct topological photonic modes. The following are examples of plasmonic nanoparticles [2]: optical waveguides [3], one-dimensional (1D) photonic crystals (PhCs) [4,5,6,7], two-dimensional (2D) PhCs [8,9], and three-dimensional (3D) PhCs [10,11]. Nevertheless, due to the complex design and manufacturing, the potential applications of topological photonics in 2D and 3D topological PhCs were very limited, particularly in the fields of integrated circuits and integrated optics devices. Consequently, topological PhCs in 1D structures are preferred for the advantages of their simple design and ease of manufacture. For example, 1D topological PhCs were studied to realize the relation between the surface impedance and the bulk band geometric phases [4]. Furthermore, 1D topological PhCs have also been employed for manipulating the light–matter interaction with studying the refractive index sensor [7], realizing multiband perfect absorption with graphene-based heterostructure [12], and generation of Fano resonance with high quality-factor [5].

Due to the advantages of sensor applications, optical sensors have gained remarkable interest and popularity as promising sensors due to their particular properties, for example, immunity to electromagnetic interference, high speed and remote sensing ability, long-distance monitoring, and fast response [13]. In addition, another feature that will be focused on which is generating hybrid resonance mode due to the strong optical localization of defect mode at the topological edge area. This is understandable, as in the coupled system of the defect cavity and the left and right parts of the topological structure when the characteristic reaction time is longer than the dephasing time, it will result in hybrid resonance modes. Additionally, the hybrid-resonance system helps to explore light–matter interactions due to its unique energy transfer and larger modifications [7]. Inspired from the previous discussion, a unique 1D topological PhC structure can be designed with strong topological properties for enhancing the hybrid resonance mode in the fulfillment of all sensing abilities and performance, such as small pulse-width, 100% transmittance, high-quality factor (Q), and a high sensitivity (S) compatible with an ultra-high figure of merit (FOM). In contrast to previous studies that have a weakness in one of the previously mentioned factors in different techniques, e.g., [14] proposed a subwavelength grating metamaterial racetrack ring resonator (SGMRTR) to enhance the strength of coupling between the ring resonator and the bus waveguide with S about 429.7 nm/RIU and Q=9800; [15] proposed a capsule-shaped in 2D PhC for high sensitivity about S = 546.72 nm/RI, but Q=2066.24 and a transmittance of 97%; [16] proposed a 2D 2W PhC with irregular slot of steeple-house, S solely about 244.42 nm/RIU; [6] proposed 1D of porous silicon structure with ultra-high sensitivity about 4784.04 nm/RIU but with Q=2149.27 and $FOM=1477.54{\;\mathrm{RIU}}^{-1}$; [7] proposed a conventional 1D topological PhC heterostructure for manipulating light–matter interaction with S=254.5 nm/RIU and Q-factor larger than 700 and FOM larger than 250.

In this paper, our proposed structure is based on two 1D topological PhCs to realize the hybrid resonance mode due to the strong optical localization at the topological edge area with high sensor abilities. According to our knowledge, it is the first time to propose a 1D topological PhC mirror and achieve higher values for all features of sensor parameters. By an appropriate design of two-1D conventional PhC for possessing photonic-band gap (PBG) in the same range of frequencies to excite a topological edge state mode when the 1D topological PhC is formed. Subsequently, the 1D-topological PhC-mirror heterostructure can be constructed when a 1D-topological PhC heterostructure and its mirror-image structures are stacked up. Accordingly, a topological mirror edge-state-mode (hybrid resonance mode) at the heterostructure interface between the two topological (original and its image) PhCs is foreseeable to appear. Then, an electro-optical (EO) polymer layer will be inserted at the interface to enhance the optical tunneling with a reduction in the pulse width, which leads to higher spatial pulse compression, and at all $n_{p}$ values, the transmission peak is almost 100% with high sensitivity. We predict the 1D topological PhC mirror heterostructure will be an outstanding idea for optical devices.

## 2. Structure and Theory

**Figure 1:**
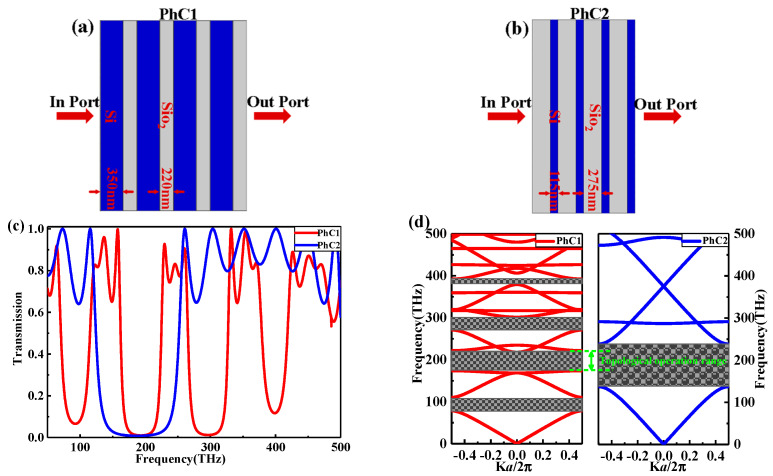
(**a**,**b**) Schematic diagrams of the proposed PhC1 and PhC2 consisting from four alternative layers of $n_{Si}=3.48\;\mathrm{and}\;n_{SiO_{2}}=1.45\;$with $d1_{Si}=350\;\mathrm{nm},\;d1_{SiO_{2}}=220\;\mathrm{nm}$ and $d2_{Si}=115\;\mathrm{nm},$ $d2_{SiO_{2}}=275\;\mathrm{nm}$, respectively; (**c**) the transmission spectrum of PhC1 and PhC2; (**d**) the band diagrams of PhC1 and PhC2.

The main topological structure of PhC is based on two PhCs, namely PhC1 and PhC2 as shown in Figure 1a,b, respectively. PhC1 is composed of four alternative layers from silicon (Si) and silicon dioxide (SiO_2_), with layer thicknesses of $d1_{Si}=350\;\mathrm{nm},\;\mathrm{and}\;d1_{SiO_{2}}=220\;\mathrm{nm}$, respectively. The transmission spectrum of the transverse electric (TE) through the proposed PhC structures is calculated by the commercial package solver of COMSOL Multiphysics using the finite element method (FEM). Whereas the top and bottom parts of the proposed structure are surrounded by a perfectly matched layer (PML) as a boundary condition to absorbing EM waves scattering. In our calculations, the refractive indices of Si and SiO_2_ are $n_{Si}=3.48\;\mathrm{and}\;n_{SiO_{2}}=1.45$, respectively. The transmission spectrum of PhC1 and PhC2 is shown in Figure 1c. From the red spectrum of PhC1, we can select the central frequency as 187.5 THz, which corresponds to the central operating wavelength $\lambda_{c}\;$of 1600 nm.

For any two materials that possess refractive indices $n_{1}$ and $n_{2}$ with thicknesses $d_{1}$ and $d_{2}$, respectively, the normal incidence gap is maximized when $n_{1}d_{1}=n_{2}d_{2}$ and the central gap wavelength is $\lambda_{c}=4n_{1}d_{1}$ $=4n_{2}d_{2}$, which means that the individual layers are exactly a quarter wavelength in thickness [17]. Based on the previous explanation, for PhC2 to get a maximum photonic bandgap (PBG) with a central wavelength of $\lambda_{c}=1600\;\mathrm{nm}$, the thicknesses of the two layers of PhC2 should be $d_{2Si}=\frac{\lambda_{c}}{4n_{Si}}=115\;\mathrm{nm}$ and $d_{2SiO_{2}}=\lambda_{c}/4n_{SiO_{2}}=275\;\mathrm{nm}$ and composed from four alternative layers from silicon dioxide (SiO_2_) and silicon (Si). The transmission spectrum of PhC2 is shown by the blue curve in Figure 1c. From the transmission spectrum, the second PBG of PhC1 is fully contained in the PBG of PhC2. It is pointed out that the topological edge modes can exist in the 1D PhC heterostructure interface when two PhC whose bandgaps are in the same wavelength range have different topological properties [18]. We can expect the topological edge modes if we stack PhC1 and PhC2. The results can be validated by calculating the band diagrams of PhC1 and PhC2, as shown in Figure 1d based on the plane-wave expansion method [18]. From Figure 1d, we can observe that the position of the second band gap from PhC1 coincides with involving with that of PhC2, which is located between the operating frequencies of 175 and 200 THz. When the two PhCs are stacked, a defect mode will appear in the range between the two green dash lines. This defect is caused by the topological operation rather than that caused by removing layers or changing the radius or dielectric constant of some layers. This confirms the possibility of the existence of the topological edge mode at the interface in this range. More clearly, there may emerge a transmission peak at the interface of the PhC heterostructure within this range of frequencies [17].

## 3. Results and Analysis

### 3.1. Topological and Topological Mirror Edge States

**Figure 2:**
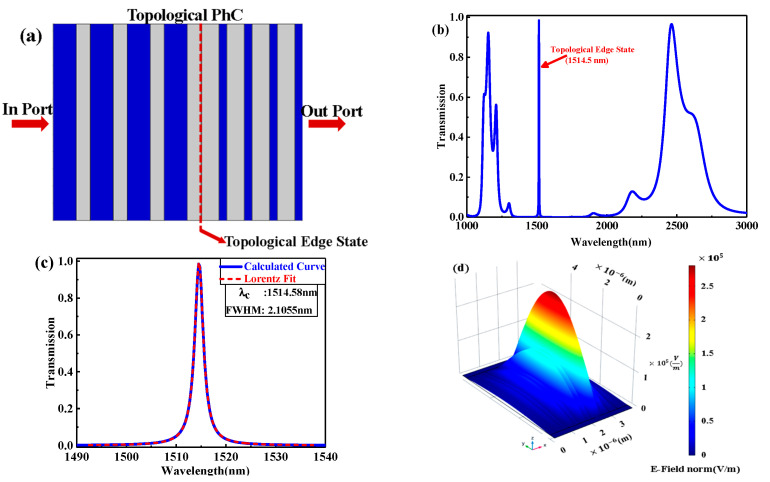
(**a**) Schematic diagram of the proposed 1D topological PhC; (**b**) the transmission spectrum of the topological PhC; (**c**) the zoom-in of the sharp peak of topological edge state and the dashed line shows the fitting curve, which is symmetric, in the typical Lorentzian-line shape; (**d**) the electric field distribution at central wavelength $\lambda_{c}=1514.58\;\mathrm{nm}$.

Figure 2 shows a schematic diagram of the proposed topological PhC as a result of stacking PhC1 and PhC2 from left to right. The transmission spectrum of the topological PhC is shown in Figure 2b, from which the topological edge state is observed in the involved range between the second PBG of PhC1 and the first one of PhC2. The zoom-in of the sharp peak of the topological edge state is shown in Figure 2c, and the dashed line shows the fitting curve, which is symmetric in the typical Lorentzian-line shape. This validates that the Lorentzian resonance was created at the interface of the topological structure [17], with 98.5% transmittance at the central wavelength $\lambda_{c}=1514.58\;\mathrm{nm}$, equivalent to central frequency $v_{c}=198\;\mathrm{THz}$, and the full width half maximum is FWHM(Δλ)=2.1055 nm. Based on the quality factor Q definition, which is the ratio of resonance frequency to FWHM(Δλ), $Q=\lambda_{c}/FWHM\left(\Delta \lambda \right)$, Q is 719.34 for the topological edge mode. In fact, the $\lambda_{c}\;$of the sharp transmission peak is exactly located at the interface point between the two PhCs, which can be shown by calculating the electric field distribution according to Figure 2d, indicating the existence of the topological edge state with the localized electric field around the topological PhC interface.

**Figure 3:**
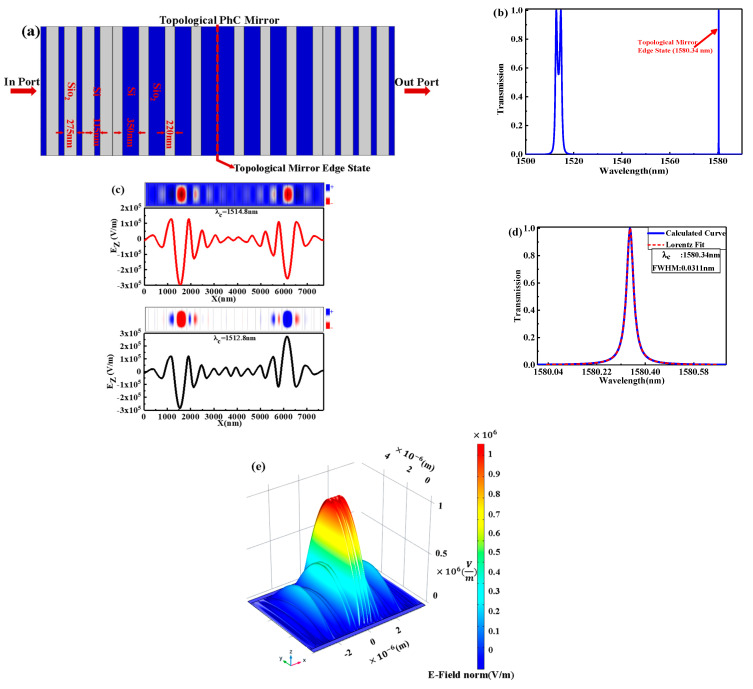
(**a**) Schematic diagram of the proposed 1D topological PhC mirror heterostructure; (**b**) the transmission spectrum of the topological PhC mirror heterostructure; (**c**) the calculated electric field *E* of z component in 1D topological PhC mirror heterostructure with mode symmetry; (**d**) The zoom-in of the sharp peak of the topological mirror edge state and the dashed line shows the fitting curve, which is symmetric, in the typical Lorentzian-line shape with 100% transmittance at central wavelength $\lambda_{c}=1580.34\;\mathrm{nm}$ and FWHM(Δλ)=0.0311 nm; (**e**) the electric field distribution in which the electric field is highly localized around zero point is exactly located at the interface point between the two topological PhCs at a central wavelength $\lambda_{c}=1580.34\;\mathrm{nm}$.

If the image view of the proposed topological PhC in Figure 2a is stacking with its original one, the topological PhC mirror heterostructure is constructed as shown in Figure 3a. Based on the previous theory, it is expected to appear in topological mirror edge-state-mode (hybrid resonance mode) at the heterostructure interface between the two topological (original and its image) PhCs. The transmission spectrum of the topological PhC mirror heterostructure is shown in Figure 3b, where two peaks of the topological edge state are observed. Meanwhile, Figure 3c shows the calculated electric field E of *z* component in 1D topological PhC mirror heterostructure with mode symmetry. In addition, $\lambda_{c}=1512.8\;\mathrm{nm}$ shows an antisymmetric mode, while $\lambda_{c}=1514.8\;\mathrm{nm}$ is a symmetric mode for *z*-polarization. Moreover, there is an interesting peak that appeared in topological mirror edge state mode. The zoom-in of the sharp peak of the topological mirror edge state is shown in Figure 3d, and the dashed line shows the fitting curve, which is symmetric, in the typical Lorentzian-line shape with 100% transmittance at central wavelength $\lambda_{c}=1580.34\;\mathrm{nm}$ and FWHM(Δλ)=0.0311 nm, corresponding to $Q=5.08\times 10^{4}$. The high Q-factor is due to the strong optical localization of the defect mode at the topological edge area. Figure 3e shows the electric field distribution in which the electric field is highly localized around the zero point, i.e., exactly at the interface point between the two topological PhCs. In addition, the E-field value is higher when compared with the case of conventional topological PhC with the same propagation length.

**Figure 4:**
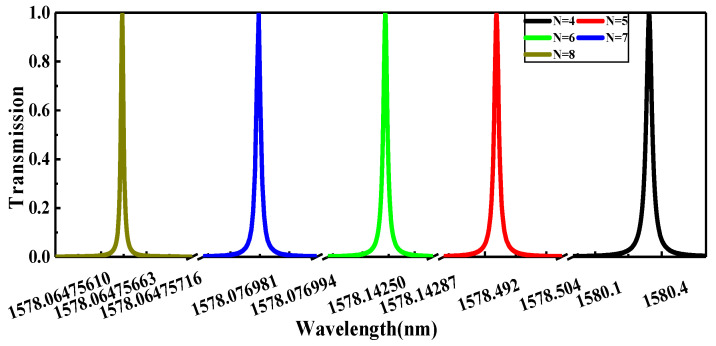
The transmission spectrum of the topological PhC mirror heterostructure for a different number of periods *n*.

With increasing the number of alternative layers, a higher Q-factor can be obtained on the account of transmittance. The structure shown in Figure 3a of the topological PhC mirror heterostructure may enhance the transmission significantly with a high Q-factor. Furthermore, Figure 4 shows the transmission spectrum of the topological PhC mirror heterostructure for a different number of periods *n*. With the increasing number *n*, the transmittance is still almost 100% due to the strong optical localization of the defect mode at the topological edge area, but with extremely fine meshing and a small step size. The curves, which are symmetric, are fitted in the typical Lorentzian-line shape and the date have been transfered into a table. Table 1 shows the Q-factor, *FWHM,* and resonance wavelength of the topological mirror edge state with the number *n*. With the increasing number *n*, *FWHM* decreases pointedly, while the resonance wavelength changes slightly, which significantly improves the Q-factor. It should be noted that increasing *n* will increase the manufacturing difficulty and cost; accordingly, we use the smaller *n* =4 under the good performance hypothesis.

By considering the structure shown in Figure 3a of topological PhC mirror heterostructure, this feature can be explained as the heterostructure interface between the two topological PhCs, the original and its image, forms a cavity with two topological PhCs on either side of the cavity as if connecting two waveguides based on photonic crystal theory so that resonance can take place at the interface. The presence of the resonance peak adapts with intuition: close to the resonant frequency, light from the input-left topological PhC (the waveguide) can couple with the cavity at the topological mirror edge area, and the cavity in sequence can couple with the output-right topological PhC (waveguide). So, the transmission peak is exactly 100%, corresponding to a strong coupling in the waveguide-cavity-waveguide system at the wavelength 1580.34 nm and generating a hybrid resonance mode. Consequently, this device acts as a narrow-band filter. Thus, the proposed topological PhC mirror has a potential application in optical fields, such as filtering, switching, and sensing.

### 3.2. Sensing Performance of Topological PhC Mirror

As mentioned above, the topological PhC mirror exhibits strong light confinement/localization at the topological mirror edge area, which gives strong intensification to the optical wave with a hybrid resonance mode wavelength that is vastly sensitive to refractive index (RI) perturbation attributed to the medium through inserting defect layer at the heterostructure interface. In a more precise context, the electro-optic (EO) polymer materials are broadly researched for use at optical communications [19], which exhibited an ample higher electro-optical coefficient and faster response times [20,21].

**Figure 5:**
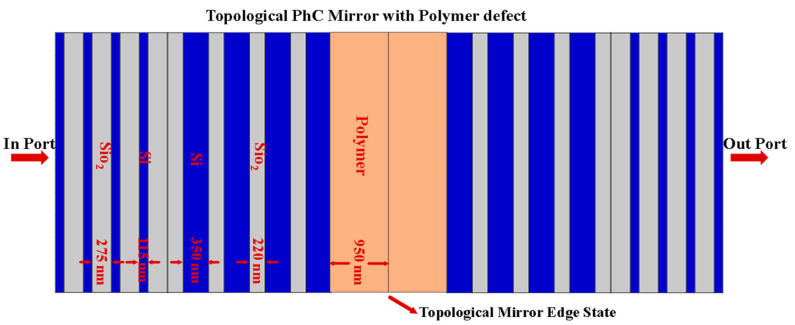
Schematic diagram of the proposed topological PhC mirror heterostructure with EO polymer defect layer.

**Figure 6:**
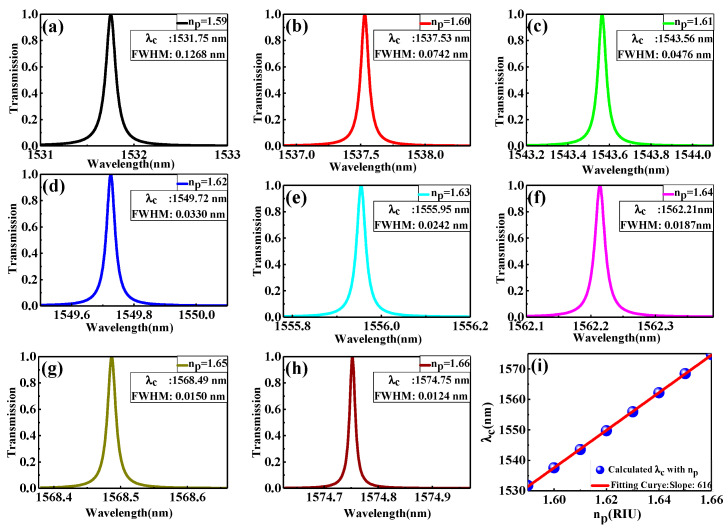
(**a**–**h**) The transmission spectrum as a function of the resonance wavelength due to changing EO polymer refractive index $n_{p}$ from 1.59 to 1.66 with an increment of $\Delta n_{p}=0.01$; (**i**) the resonance wavelength shifting Δλ as a function of $n_{p}\;$ variation where the simulated data is characterized by the blue sphere and the linear fitting by the red solid line.

In this context, the EO sensor based on the proposed topological PhC mirror can be studied by inserting the EO polymer defect layer at the interface with thickness $d_{p}$ fixed at 950 nm. The choice of this thickness is due to two reasons. The first is to operate the sensor around the optical communication wavelength. Second, at this thickness, there is a highly defect resonance mode that enhances the interaction between the light and sensing medium. In addition, the EO polymer possesses a tunable index of refraction between 1.59 and 1.66 [22]. Figure 5 shows a schematic diagram of the proposed topological PhC mirror heterostructure with the EO polymer defect layer. Figure 6a–h displays the transmission spectrum as a function of the resonance wavelength due to the changing of the EO polymer refractive index $n_{p}$ from 1.59 to 1.66 with an increment of $\Delta n_{p}=0.01$. As shown in these figures, all the resonance wavelengths shift in the direction of longer wavelength (i.e., redshift) from 1531.75 nm to 1574.75 nm around the optical communication wavelength as a function of the increasing $n_{p}$ with the decreasing *FWHM* from 0.1268 nm to 0.0124 nm.

Thus, introducing the EO polymer defect layer at the interface caused strong optical localization of the defect mode at the topological edge area enhanced the light–matter interaction with reducing pulse width, which leads to higher spatial pulse compression, and at all $n_{p}$ values, the transmission peak is almost 100%. In sensor devices, the sensitivity (S), the quality factor (Q), and the figure of merit (FOM) are imperative indicators for appraising the sensing ability and performance. S measures the shift of the resonance wavelength Δλ produces during the change in the refractive index Δn and can be expressed as S=Δλ/Δn by the unit of nm/RIU. The FOM can be defined as the ratio of S to FWHM, FOM=S/FWHM, by the unit of ${\mathrm{RIU}}^{-1}$. The Q was defined previously as $Q=\lambda_{c}/FWHM$. To distinguish the sensor parameters, the resonance wavelength shifting is detected as a function of $n_{p}$, as shown in Figure 6i, where the simulated data is characterized by the blue sphere and the linear fitting by the red solid line. From linear fitting, S (the slope) is 616 nm/RIU. The sensing capabilities are summarized in Table 2. The high Q values make the accurate sensor capacities possible and improve the wavelength resolution [23]. In addition, the FOM and Q control the performance and efficiency of sensors [6]. Accordingly, we accomplished a high sensing ability. The Q values reached $10^{5}$, which is an ultra-high-quality factor, and FOM is extremely high at about $49,677.42{\;\mathrm{RIU}}^{-1}.$

## 4. Conclusions

In conclusion, a new type of 1D topological photonic crystal (PhC) heterostructure is introduced, namely, a 1D topological photonic crystal (PhC) mirror heterostructure. In the proposed heterostructure, the electric field profile is highly localized around zero point, i.e., exactly at the interface point between the two topological PhCs. The topological edge-area mode has existed with the validation of Lorentzian resonance at the mirror heterostructure interface with 100% transmittance. In addition, FWHM(Δλ)=0.0311 nm corresponding to $Q=5.08\times 10^{4}$. Moreover, introducing the EO polymer defect layer at the interface with an increasing $n_{p}$, the resonance wavelength changes from 1531.75 nm to 1574.75 nm around the optical communication wavelength as a function of the increasing $n_{p}$ with the decreasing FWHM from 0.1268 nm to 0.0124 nm with strong optical tunneling of the defect mode at the topological edge area. Finally, we accomplished a high sensitivity of about 616 nm/RIU with a Q value of 126,995.97, which is a high-quality factor, and a high FOM of about $49,677.42{\;\mathrm{RIU}}^{-1}.$

## Figures and Tables

**Table 1:** Q -factor, *FWHM,* and resonance wavelength of the topological mirror edge state with the number of periods *n*.

N	$\lambda_{c}\left(nm\right)$	FWHM(nm)	Transmittance %	Q
4	1580.34352	0.03111	100	$5.079\times 10^{4}$
5	1578.49427	0.00106	99.98	$1.49\times 10^{6}$
6	1578.14266	$3.457\times 10^{-5}$	99.98	$4.56\times 10^{7}$
7	1578.07699	$1.111\times 10^{-6}$	99.97	$1.42\times 10^{9}$
8	1578.06476	$3.568\times 10^{-8}$	99.95	$4.42\times 10^{10}$

**Table 2:** The summary of sensing capabilities.

$n_{p}$	$\lambda_{c}\;\left(nm\right)$	FWHM(nm)	Transmittance %	Q	$FOM\;\left(RIU^{-1}\right)$
1.59	1531.75	0.1268	100	12,080.05	4858.04
1.60	1537.53	0.0742	100	20,721.43	8301.88
1.61	1543.56	0.0476	100	32,427.73	12,941.17
1.62	1549.72	0.0330	99.98	46,961.21	18,666.67
1.63	1555.95	0.0242	100	64,295.45	25,454.55
1.64	1562.21	0.0187	100	83,540.64	32,941.18
1.65	1568.49	0.0150	99.89	104,566.00	41,066.67
1.66	1574.75	0.0124	99.91	126,995.97	49,677.42
